# Urinary organic acid analysis in preventive health assessment and healthy-aging research: A state-of-the-art review of evidence and methodological limitations

**DOI:** 10.1016/j.metop.2026.100511

**Published:** 2026-10-15

**Authors:** Markus Schauer, Susanne Mair, Mohamad Motevalli, Florian Kainz, Katharina Wirnitzer

**Affiliations:** a Limbach Research Institute for Preventive Diagnostics and Healthy Aging, German University of Health & Sport (DHGS), Ismaning, 85737, Germany; b Department of Sport Science, German University of Health & Sport (DHGS), Ismaning, 85737, Germany; c Department of Sport Science, University of Innsbruck, Innsbruck, 6020, Austria; d Department of Pediatric Oncology and Hematology, Otto-Heubner Centre for Paediatric and Adolescent Medicine (OHC), Charité – Universitätsmedizin Berlin, Berlin, 13353, Germany; e Charité Competence Center for Traditional and Integrative Medicine (CCCTIM), Charité – Universitätsmedizin Berlin, Berlin, 13353, Germany; f Department of Secondary Education, University College of Teacher Education Tyrol, Innsbruck, 6010, Austria

**Keywords:** Urinary organic acids, Citrate, Succinate, Hippurate, Methylmalonic acid, Urinary metabolomics, Diagnostics, Aging, Longevity, Health monitoring

## Abstract

Urinary organic acid (UOA) testing has established diagnostic applications in inherited metabolic disorders, but its relevance to preventive health assessment and healthy-aging research requires critical evaluation. This state-of-the-art review examines adult human evidence while distinguishing clinical organic acid testing, commercial panels, and targeted or untargeted urinary metabolomics.

Eight complementary PubMed searches, supplemented by targeted reference-list checking, yielded 3054 unique records for title and abstract screening. Data from 38 human-study reports, including population characteristics, analytical methods, metabolites, outcomes, confounder adjustment, evaluation procedures, and study limitations, were extracted and synthesized narratively. Analytical and normalization strategies, interpretative limitations, and multi-omics approaches were critically appraised, distinguishing biological associations and exploratory prediction from validated clinical utility.

Across the reviewed studies, urinary metabolite profiles were associated with adiposity, metabolic syndrome, renal outcomes, microbial features, and aging-related phenotypes. Evidence ranged from small cross-sectional investigations to population cohorts and prospective analyses, with independent-cohort replication of selected associations and external evaluation of selected predication models. Prospective findings concerned specific populations and outcomes, whereas aging-related evidence included both positive and null findings. Renal handling, sampling conditions, normalization, and analytical heterogeneity complicated comparisons across studies. Multi-omics investigations supported biological interpretation and exploratory prediction, but the reviewed studies did not establish that UOA-guided assessment improves preventive or healthy-aging outcomes.

UOA testing has established diagnostic value in inherited metabolic disorders, while emerging human evidence supports further investigation of its relevance to preventive health assessment and healthy-aging research. Translation into routine care requires assay-specific validation and evidence that test-guided assessment or intervention improves clinically meaningful outcomes.

## 1 Introduction

Advances in preventive health assessment increasingly emphasize the need for biomarkers capable of capturing functional metabolic alterations before the manifestation of overt disease [1], [2]. In this context, metabolomics investigates metabolic changes associated with genetic predisposition, environmental exposures, nutrition, lifestyle, and host–microbiome interactions [2], [3].

As part of these metabolic profiling approaches, the analysis of organic acids in urine has been proposed as a functional readout of intermediary metabolism. Urinary organic acids represent end products and intermediates of multiple biochemical pathways, including carbohydrate metabolism and glycolysis (e.g., lactate, pyruvate), amino acid catabolism (e.g., phenylpyruvate, α-ketoisovalerate), fatty acid β-oxidation and lipid metabolism (e.g., adipate, suberate), and mitochondrial energy metabolism within the tricarboxylic acid (TCA) cycle (e.g., citrate, succinate, fumarate, malate), as well as microbial-derived or host–microbiome co-metabolites (e.g., hippurate, p-hydroxybenzoate) [4]. Chromatographic–mass spectrometric methods are used to measure urinary organic acids, with gas chromatography–mass spectrometry (GC-MS) widely employed in clinical testing for inherited metabolic disorders [5]. Foundational work describing the composition and diversity of the human urine metabolome has provided an essential basis for subsequent applications of urinary metabolomics in human research [6].

Urinary organic acid (UOA) testing has established diagnostic value in inherited metabolic disorders [5], [7]. Beyond these indications, human studies have reported associations between urinary metabolites and metabolic phenotypes, including metabolic syndrome [8]. These findings have encouraged interest in UOA analysis for preventive health assessment, supported by the non-invasive nature of urine collection and the feasibility of repeated sampling for research [9]. Associations involving selected urinary organic acids provide a basis for further biomarker investigation [8]. However, such associations still do not establish that urinary organic acid profiling can identify subclinical dysfunction, guide individualized prevention, or monitor metabolic resilience in routine care. Nor do they demonstrate earlier detection than conventional blood tests or identify a clinically defined state of “metabolic vulnerability.” Interpretation of urinary metabolomic measurements requires attention to pre-analytical conditions [10] and normalization strategy [11], while translation of exploratory findings into clinical tests requires appropriate validation [12]. Table 1 summarizes the analytical capabilities and levels of evidence for urinary organic acid profiling in preventive and healthy-aging contexts, distinguishing supported interpretations from their evidentiary boundaries.

**Table 1:** Analytical capabilities and levels of evidence for urinary organic acid profiling in preventive and healthy-aging contexts.

Evidence category	Supported interpretation	Boundary of interpretation
Analytical capability	Detection and, where analytically validated, quantification of specified urinary metabolites; non-invasive collection permits repeated sampling.	Measurement capability and repeat sampling do not establish clinical usefulness for longitudinal monitoring.
Biological plausibility	Associations with biochemical pathways provide hypotheses about metabolic processes.	Urinary profiles do not directly establish pathway dysfunction, micronutrient insufficiency, or metabolic vulnerability.
Exploratory research applications	Investigation of associations with metabolic health, aging-related outcomes, within-person variation, and other omics measurements.	Findings apply to the specific assay and study population; associations do not establish causality or benefit from test-guided interventions.
Clinically validated utility	Clinical usefulness must be demonstrated for a defined assay, population, and intended application.	The evidence summarized here does not establish improved preventive or healthy-aging outcomes from commercial UOA-panel-guided care.

Against this background, a balanced, evidence-based appraisal of UOA analysis is warranted. The present review differentiates among clinical organic acid testing for inborn errors of metabolism, commercial organic acid panels marketed for functional or preventive assessment, targeted quantitative urinary metabolomics, and untargeted urinary metabolomics. Throughout this review, evidence concerning a particular assay, panel, or metabolomic workflow is interpreted within its specific analytical and clinical context. Shared metabolites or instrumentation do not establish equivalent validation or clinical utility. A recent review describes the historical development, analytical workflows, and clinical interpretation of UOA testing, principally in the diagnosis of inherited metabolic disorders [5]. The present review complements that account through a study-level critical synthesis of adult human evidence concerning urinary organic acids and related urinary metabolomic profiles beyond inherited disorders. Its contribution is to appraise these findings within explicit distinctions between analytical capability, biological plausibility, exploratory research, and clinically validated utility. Particular attention is given to whether reported associations, prospective findings, and multi-omics results justify proposed preventive or aging-related applications.

## 2 Methods

The present research is a state-of-the-art narrative review of UOA measurements and related urinary metabolomic evidence, including their potential relevance to preventive and aging research. A narrative approach was selected to integrate heterogeneous clinical and methodological evidence, discuss current developments, and identify knowledge gaps and future research directions [13], [14], [15].

### 2.1 Literature search

A multi-stage literature search was conducted in PubMed, with the final searches performed in September 2026. PubMed was selected for its broad biomedical and clinical coverage and reproducible search functionality, with targeted reference-list checking used to identify additional relevant publications. Eight complementary search strategies addressed urinary organic acid analysis, urinary metabolomics and aging, cardiometabolic associations, selected organic acid metabolites, multi-omics integration, analytical and preanalytical methods, and longitudinal clinical outcomes. Free-text terms and, where applicable, Medical Subject Headings were combined using Boolean operators OR for alternative terms and for intersecting concepts. The searches covered all publications up to the search date, without any minimum year restriction. No language, age, or study-design filters were applied during retrieval; these restrictions were considered during eligibility assessment. Complete search strings, field tags, PubMed query translations, and results per search are provided in Supplementary Table S1.

Supplementary identification involved PubMed-based checks of references in the original manuscript and targeted examination of the reference lists of six relevant publications. The latter encompassed 440 reference entries, from which keyword-based prioritization identified 54 distinct linked PubMed records for retrieval. Restriction to PubMed may have resulted in omission of relevant studies indexed elsewhere. Nevertheless, eight complementary, topic-specific search strategies and targeted reference-list checking supported coverage of the review's principal themes.

### 2.2 Eligibility criteria

Eligible publications comprised peer-reviewed human research, systematic reviews, methodological studies, and multi-omics analyses relevant to urinary organic acids in metabolic health, preventive health assessment, or aging. Publications were eligible if they presented quantitative metabolite data, pathway-based interpretations, associations with clinical or functional outcomes, or methodological evaluations of analytical platforms, normalization procedures, or data-integration approaches. Studies were excluded if they involved animal models, focused exclusively on pediatric samples, were available only as conference abstracts, were commentaries or opinion pieces, or were published in a language other than English. Primary human studies provided the basis for study-level clinical findings, while systematic reviews and methodological publications provided contextual and methodological evidence.

### 2.3 Record management, study selection, and data extraction

A total of 3641 records were identified before removing duplicates. After eliminating 642 duplicate occurrences across searches, the dataset comprised 2999 unique records. Checks of the original bibliography and selected reference lists contributed 55 additional unique records, resulting in a combined total of 3054 records.

Titles and abstracts were screened by MM. Publications deemed potentially eligible were assessed in full by MM and MS, and any uncertainties were resolved through discussion. In total, 146 publications underwent full-text review, of which 38 human studies met the inclusion criteria. Principal reasons for non-inclusion in the study-level synthesis were the absence of relevant urinary measurements, populations or clinical topics outside the review's scope, and ineligible publication types. Reviews and other publications used solely for contextual discussion were not counted among the 38 human-study reports. The selection process is summarized in Fig. 1.

**Fig. 1:**
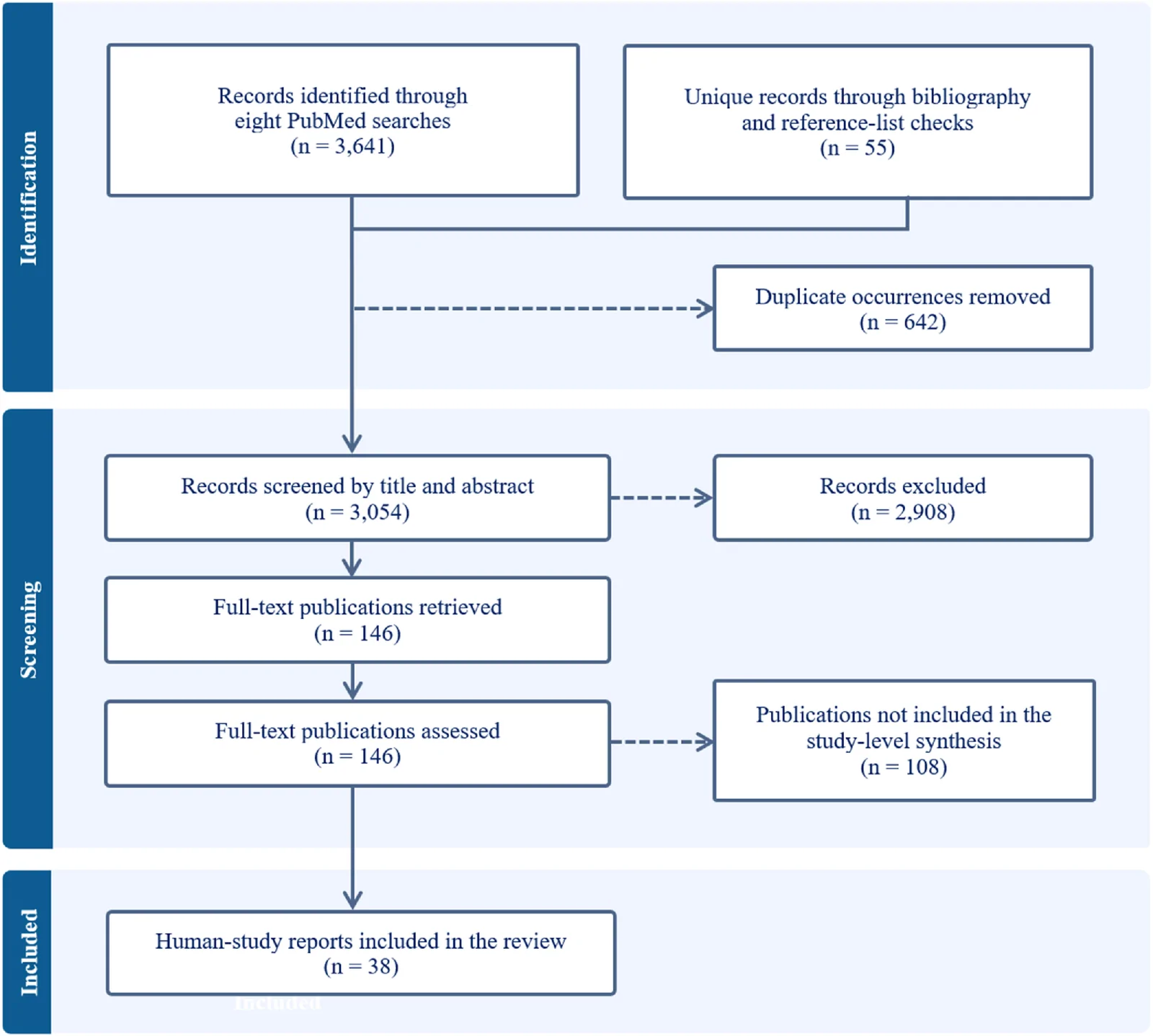
Flow diagram of record identification, screening, full-text assessment, and inclusion in the study-level evidence synthesis.

Extracted information included report details; study design and population characteristics, including sample size and available age information; sample collection and analysis, including analytical methods and normalization; organic acids and related markers; principal findings, including clinical or functional outcomes and reported effect estimates; confounder adjustment and evaluation procedures; and major limitations. Study-level information was extracted by MM and checked against the original publications by MS. Verification covered study characteristics, analytical methods, numerical findings, confounder adjustment, evaluation procedures, and limitations. Disagreements regarding study inclusion or extracted information were resolved through discussion between the involved authors and re-examination of the original publication.

### 2.4 Narrative synthesis

Evidence was synthesized narratively and organized by topic, covering analytical methodology, biological interpretation, clinical associations, aging-related outcomes, and multi-omics integration. Evidence was interpreted according to the reported intended use, metabolite coverage, measurement strategy, and supporting evidence for each assay. Interpretation differentiated analytical performance, cross-sectional associations, longitudinal predictive value, and demonstrated clinical utility. Findings from broader urinary metabolomics studies were separated from evidence directly evaluating urinary organic acid panels. Reports were classified according to their urinary measurement strategy as targeted organic-acid measurements, broader urinary metabolomic profiling, or aggregate organic anion measurement. The use of additional omics was recorded separately, distinguishing cross-omics statistical analyses or comparisons of omics-derived scores from parallel or pathway-level investigations. Measurement of metabolites in multiple biological specimens alone was not classified as multi-omics. No standardized quality-appraisal tool was applied. Methodological quality and study limitations were appraised narratively, considering study design, confounder adjustment, analytical methods, evaluation procedures, and applicability.

## 3 Biological and analytical foundations of urinary organic acid analysis

Urinary organic acids can be investigated using qualitative, semiquantitative, or quantitative approaches, depending on the analytical objective and workflow [4]. The human urine metabolome contains compounds arising from diverse endogenous, dietary, and microbial sources [6]. Clinical testing for inherited metabolic disorders, commercial functional-medicine panels, and research metabolomics should therefore be distinguished by their intended use, metabolite coverage, reporting, validation requirements, and interpretation. Methodological standardization is essential for evaluating analytical performance and comparability [2]. Urinary concentrations or excretion rates reflect the combined effects of metabolite production, systemic metabolism, and renal handling [16]; they should not be equated directly with intracellular metabolic flux.

### 3.1 Biochemical scope and renal handling

Organic acids detectable in urine encompass intermediates of central metabolic pathways, including glycolysis, the tricarboxylic acid cycle, amino acid metabolism, fatty acid β-oxidation, and ketone body metabolism [6]. Paired urinary metabolomics and stool sequencing have identified associations between microbial features and urinary hippurate, succinate, formate, and 4-hydroxyphenylacetate [17]. Urinary metabolite concentrations reflect systemic metabolism together with renal transport and kidney function [16]. An altered urinary concentration does not, by itself, establish impaired pathway function or micronutrient insufficiency. Such interpretations require independent evidence relevant to the specific metabolite, assay, and clinical context.

Renal handling is a determinant of urinary metabolite output, not merely a potential confounder. Urinary excretion reflects the balance of glomerular filtration, tubular secretion and reabsorption, together with renal metabolism; the resulting concentration also depends on urine flow. Consequently, a change in urinary organic acid concentration cannot be attributed to altered systemic production without considering kidney function [16]. Medications can modify these processes through transporter inhibition or competition, as illustrated by human evidence that probenecid alters plasma and urinary metabolite profiles [18].

These considerations are particularly relevant in older adults. Longitudinal measurements demonstrate age-related glomerular filtration rate (GFR) decline and differences between women and men [19]. Sarcopenia-related reductions in creatinine generation and excretion can also affect interpretation: a lower urinary creatinine denominator can increase a metabolite-to-creatinine ratio without a corresponding increase in metabolite excretion. Low muscle mass can additionally cause creatinine-based estimated glomerular filtration rate (eGFR) to overestimate kidney function [20]. Thus, creatinine normalization and creatinine-based estimation of GFR address different questions but can both be influenced by muscle mass. It should be considered that hydration changes urine flow and concentration, whereas altered filtration and tubular transport change metabolite handling itself. Correcting dilution therefore does not remove the effects of kidney dysfunction, and adjustment for eGFR alone does not fully characterize tubular secretion or reabsorption. Age-associated urinary differences should consequently be interpreted alongside renal function, muscle mass, sex, hydration, and medication exposure.

### 3.2 Analytical methods, normalization, and quality assurance

GC-MS is widely used in clinical organic acid analysis for inherited metabolic disorders [4]. Research investigations may employ GC-MS, liquid chromatography–mass spectrometry (LC-MS), capillary electrophoresis–mass spectrometry (CE-MS), or nuclear magnetic resonance (NMR), with targeted or untargeted measurement strategies. Instrument choice alone does not establish metabolite coverage, quantitative accuracy, or fitness for clinical use, and methodological standardization remains essential for comparability [2]. For targeted quantitative assays, validation should address the intended analytes, calibration, selectivity, precision, accuracy, reportable range, and relevant matrix effects [21]. Untargeted workflows require documented quality control, analytical reproducibility, and transparent reporting of metabolite identification or annotation [22].

Urine-dilution correction should be selected according to the population, analytical platform, and study objective. Creatinine normalization is convenient but can introduce variation associated with muscle mass, age, sex, and renal function [11]. Specific gravity provides a practical alternative, although its interpretation can be affected by urinary composition, including glucose and protein [23]. Osmolality measures total dissolved particle concentration and can support pre-acquisition dilution adjustment; however, altered solute excretion and kidney disease can affect its suitability as a normalization reference [24]. Twenty-four-hour excretion estimates metabolite output over a defined interval rather than concentration alone, but depends on accurate timing and complete urine collection.

Post-acquisition methods address a different aspect of variability. Probabilistic quotient normalization (PQN) estimates a sample-specific dilution factor from metabolite or spectral-intensity ratios relative to a reference profile; it assumes that most signals do not undergo coordinated biological changes [23]. Total useful signal normalization scales measurements to a defined set of retained analytical signals, but depends on feature selection and may obscure genuine changes in overall metabolite abundance [11], [24]. Internal-standard-based correction addresses analytical variation, including extraction losses and instrument response where the standards adequately represent the analytes; it does not independently correct physiological urine dilution [21]. Therefore, no normalization strategy is universally appropriate. Studies should distinguish physiological dilution correction from analytical signal correction, justify their chosen method, and assess whether principal findings are robust to reasonable alternatives. The relevance of this distinction is illustrated by an aging study, in which creatinine normalization altered observed age associations [25].

Metabolite identification confidence should be reported separately from measurement precision. Under the Metabolomics Standards Initiative framework, confirmed identification requires comparison with an authentic standard analyzed under identical experimental conditions using at least two independent, orthogonal properties; library-based annotations without such confirmation should be distinguished from confirmed identities [26]. For quantitative assays, authentic calibrators and suitable internal standards should support assessment of calibration range, accuracy, precision, limits of detection and quantification, extraction recovery, stability, and matrix effects, as appropriate to the method and intended use [21]. Detection alone does not establish reliable quantification, and matrix-dependent ion suppression or enhancement requires particular attention in LC-MS assays [21].

Internal quality control (QC) should include appropriate blanks, system-suitability checks, and repeated QC samples. Pooled study QC samples can monitor reproducibility and drift in untargeted workflows, whereas quantitative assays require controls with assigned concentrations across the relevant measurement range [21], [22]. Sample order and batch allocation should minimize confounding with biological groups, and any drift or batch correction should be documented and evaluated to avoid removing genuine biological variation [22]. External quality assessment or interlaboratory proficiency testing provides an additional check beyond within-run reproducibility, as demonstrated for clinical urinary organic acid analysis [27]. Inter-platform harmonization requires comparison of confirmed shared analytes, compatible units and calibration, and suitable shared reference materials; statistical batch correction alone cannot establish equivalence between GC-MS, LC-MS, CE-MS, and NMR measurements [21], [22], [26].

### 3.3 Pre-analytical considerations

Urine collection should be matched to the study objective rather than follow a universal preference for first-morning samples. Spot specimens are convenient for large cohorts, but collection time, recent food and fluid intake, and dilution require consideration. First-morning urine is a standardized spot specimen that may facilitate repeated comparisons, but reflects overnight accumulation and does not eliminate dietary or circadian effects [28], [29].

Timed collections capture excretion over a defined interval and are useful when investigating responses to meals, interventions, or other short-term exposures; serial collections at specified times are needed to characterize within-day variation [30]. Complete 24-h collections permit estimation of daily metabolite excretion when concentration and total volume are measured, but increase participant burden, are vulnerable to incomplete collection, and obscure within-day fluctuations. Their suitability also depends on maintaining metabolite stability throughout collection and storage [10].

Accordingly, standardized spot or first-morning samples may be appropriate for comparisons of metabolite profiles, timed or serial samples for exposure-related and temporal responses, and complete 24-h collections for daily excretion. The protocol should document collection timing and duration, dietary and hydration conditions, completeness where relevant, processing delays, storage temperature, preservatives where used, and freeze–thaw history. These procedures should be validated for the target metabolites and analytical platform [10].

### 3.4 Clinical organic acid testing, commercial panels, and research metabolomics

Clinical organic acid testing for inherited metabolic disorders is directed toward recognizing diagnostically informative biochemical abnormalities and interpreting them alongside clinical findings and complementary investigations [4]. Its established diagnostic role should be distinguished from preventive applications in otherwise healthy adults.

Commercial panels marketed for functional or preventive assessment require panel-specific appraisal. Metabolite coverage, analytical validation, quality-control procedures, reference intervals, and interpretative claims should be evaluated for the particular laboratory method and intended use. Reliable measurement of an individual metabolite does not, by itself, validate interpretations concerning micronutrient requirements, mitochondrial dysfunction, microbial dysbiosis, or the benefit of panel-guided interventions. Targeted quantitative metabolomics measures predefined analytes using an explicitly characterized assay, whereas untargeted metabolomics surveys a broader range of detectable features for discovery and metabolic characterization. Validation requirements depend on the measurement objective and intended application [21], [22]. Findings from either research approach should not be transferred to a commercial panel without establishing analytical comparability and validating the proposed clinical interpretation. Table 2 summarizes the analytical objectives, measurement requirements, and interpretation of these approaches.

**Table 2:** Distinctions between clinical organic acid testing, commercial panels, and urinary metabolomics approaches.

	Main objective and coverage	Measurement and quality requirements	Reference intervals and interpretation
**Clinical organic acid testing for inherited metabolic disorders**	Identify diagnostically informative organic acid patterns; coverage follows the clinical workflow.	Commonly GC-MS profiling; method validation, laboratory QC, and diagnostic interpretation appropriate to the assay.	Interpret biochemical patterns and laboratory findings within clinical context; diagnostic evidence applies to the relevant inherited disorders.
**Commercial functional or preventive panels**	Assess a provider-defined metabolite panel with associated functional interpretations.	Evaluate the specific assay, quantitative performance, QC, and reporting procedures; do not infer equivalence from shared instrumentation.	Evaluate the derivation of reference intervals and supporting evidence for each interpretative claim and intended application.
**Targeted quantitative urinary metabolomics**	Measure predefined metabolites for specified research or clinical questions.	Analyte-specific calibration and validation appropriate to intended use; documented precision, accuracy, selectivity, and quantitative range.	Quantitative measurements and research reference distributions do not automatically establish clinical decision limits or utility.
**Untargeted urinary metabolomics**	Discover associations across a broad range of detectable metabolites or features.	GC-MS, LC-MS, CE-MS, or NMR workflows; QC, reproducibility, annotation confidence, and appropriate statistical validation.	Feature differences or research signatures require further validation before use as clinical tests or transfer to another assay.

Abbreviations: CE-MS, capillary electrophoresis–mass spectrometry; GC-MS, gas chromatography–mass spectrometry; LC-MS, liquid chromatography–mass spectrometry; NMR, nuclear magnetic resonance; QC, quality control. Categories distinguish intended use and analytical strategy and can overlap technically. The quality requirements listed are appraisal criteria, not assurances that every study or commercial assay satisfies them. Analytical platforms alone do not determine whether a workflow is targeted, untargeted, or clinically validated [4], [21], [22].

### 3.5 Interpretation criteria

Reference intervals and interpretative criteria must be considered within the relevant modality. Clinical organic acid testing for inherited metabolic disorders integrates laboratory findings with characteristic biochemical patterns and clinical context [4]. For targeted quantitative assays, reference intervals and decision limits require evaluation for the specified method, population, and intended use; a research-cohort distribution is not automatically a clinical decision threshold. Pattern recognition and biological interpretation are distinct steps in metabolomics [31]. Untargeted feature intensities and multivariate research scores should not be interpreted using reference intervals established for a different quantitative assay. Likewise, a result outside a commercial panel's reference interval does not independently validate the biological explanation or intervention attached to that result.

## 4 Human evidence

The human evidence includes targeted organic acid measurements, broader targeted quantitative metabolomics, untargeted urinary profiling, and multi-matrix or multi-omics models. These research approaches are considered separately from clinical testing for inherited metabolic disorders and commercial functional or preventive panels; their findings do not establish interchangeable evidence for a single UOA test. The available studies range from small cross-sectional comparisons to population cohorts with longitudinal outcomes. Differences in study populations, urine collection protocols, analytical platforms, normalization strategies, and clinical outcomes limit direct comparison across studies. Table 3 summarizes their populations, sampling and analytical methods, findings, adjustment, validation, and limitations.

**Table 3:** Human studies of urinary organic acids and related metabolomic profiles.

Report	Design and population	Sample collection and analysis	Organic acids and related markers	Principal findings	Adjustment and evidence assessment	Major limitations
Haam JH et al. (2021) [8]	Cross-sectional health-check cohort; 529 Korean adults: 246 men, 283 women	Targeted organic-acid measurement. HPLC-MS organic-acid profiling	Pyruvate, alpha-ketoglutarate, branched-chain ketoacids, FIGLU, quinolinate	Selected organic acids associated with MetS (age/sex-adjusted ORs 1.92–2.81); several associations attenuated after insulin-resistance adjustment.	Age/sex adjustment; additional HOMA2-IR adjustment.	Cross-sectional health-check cohort; upper-quintile values do not establish diagnostic thresholds.
Bruzzone C et al. (2021) [32]	Large cross-sectional classification study (11,754 Europeans); 41% women; ages 18–75 y	Broader urinary metabolomics. Standardized urine collection; samples stored at −80°C. NMR analysis; Pareto scaling for spectral analyses.	Quinolinic, salicyluric, methylhippuric, nicotinuric acids; 4-HPPA/p-cresyl-sulfate signal among 17 markers.	A 17-metabolite signature classified prevalent MetS (AUROC 0.83–0.87).	FDR correction and renal-function assessment; 75%/25% training–test split within the study sample; additional NAFLD cohort application.	Prevalent-disease classification; broad metabolite panel with some ambiguous NMR assignments.
Pak J et al. (2025) [33]	Cross-sectional exploratory profiling; 127 normal, borderline and MetS participants	Broader urinary metabolomics. Morning midstream urine collected after an overnight fast. GC-MS profiling identified 80 metabolites; total-sum metabolite intensity normalization.	Glucuronate, galacturonic, citramalic, pyroglutamic and 4-hydroxyphenylacetic acids; broader 80-metabolite profile.	Sugar-acid and other metabolite profiles differed across normal, borderline and MetS groups.	Multiplicity correction; PLS-DA permutation testing and ROC analysis within the study sample.	Small sample (n = 127); exploratory classification; potential overfitting and residual confounding.
Elliott P et al. (2015) [34]	Cross-sectional discovery and country validation INTERMAP; US n = 1880; UK n = 444; ages 40–59 y	Broader urinary metabolomics. Two 24-h urine collections ∼3 weeks apart; NMR and ion-exchange chromatography; 24-h metabolite excretion with additional creatinine adjustment.	Citrate, succinate, 2-hydroxyisobutyrate, hippurate and other metabolites.	Twenty-nine urinary metabolites associated with BMI; 25 replicated in the UK cohort.	Demographic, lifestyle, dietary, and clinical adjustment, plus creatinine; independent UK replication of BMI associations; repeat urine sampling.	Cross-sectional associations; causality and clinical utility remain unestablished.
Calvani R et al. (2010) [35]	Case–control pilot; 15 morbidly obese, insulin-resistant men and 10 controls; 2 postsurgical observations.	Broader urinary metabolomics. Urinary ^1^H NMR.	Hippurate and 2-hydroxyisobutyrate within a broader profile.	Obesity-associated profiles differed from controls; profiles changed in two postsurgical cases.	Two postsurgical observations; not independent cohort validation.	Small, male-only sample; two surgical observations cannot establish efficacy.
Yi X et al. (2026) [36]	Cross-sectional population study; 5831 Chinese participants.	Broader urinary metabolomics. Untargeted LC-QToF-MS; 340 candidate metabolites.	Broad metabolomic profile.	32 metabolites associated with BMI (Q < 0.01).	Age/sex adjustment; false-discovery control (Q < 0.01).	Cross-sectional; no organic-acid-only panel performance established.
Haam JH et al. (2022) [37]	Cross-sectional imaging study; 133 Korean adults (68 men, 65 women); age 58.6 ± 10.9 y.	Targeted organic-acid measurement. Overnight fasting with specified dietary/supplement restrictions; HPLC-MS; creatinine normalization.	TCA intermediates, ethylmalonate, β-hydroxybutyrate, FIGLU and p-hydroxyphenyllactate.	Fibrosis ORs: ethylmalonate 0.390 (95% CI 0.162–0.939); β-hydroxybutyrate 0.393 (0.162–0.956). Steatosis associations attenuated after metabolic adjustment.	Age/sex, ALT or FIB-4, BMI and HOMA2-IR adjustment; MRI and magnetic resonance elastography outcomes; no independent cohort.	Cross-sectional; imaging rather than biopsy; study-defined upper quartiles; residual dietary confounding.
Salihovic S et al. (2020) [38]	Two-cohort discovery/replication with prospective follow-up; 1424 older adults; follow-up ≤12 y.	Broader urinary metabolomics. PIVUS spot urine; ULSAM 24-h urine; UPLC-MS; creatinine adjustment.	Broad profile, including putatively annotated hydroxyundecanoylcarnitine and nonanoylcarnitine.	Adding six metabolites numerically increased the incident-T2D C-statistic from 0.866 to 0.892 in the held-out test sample; improvement in model fit was not statistically significant.	Demographic, creatinine and cardiometabolic adjustment; independent-cohort replication of prevalent-T2D associations. Incident-T2D model: internal cross-validation and a two-thirds/one-third training–test split of the pooled cohorts.	Different collection protocols; tentative annotations; older populations; clinical benefit untested.
Liu JJ et al. (2018) [39]	Nested case–control study in T2D; discovery 116 cases/271 controls; validation 96/402.	Targeted organic-acid measurement. Baseline urinary TCA-cycle metabolite measurement.	Lactate, pyruvate, citrate, α-ketoglutarate, succinate, fumarate and malate.	Fumarate and malate predicted rapid eGFR decline (≥5 mL/min/1.73 m^2^/y); fumarate–sex interaction p = 0.03.	Cardiorenal adjustment, including eGFR and albuminuria; replication of metabolite associations in an independent nested case–control sample.	T2D-specific renal outcome; potential overlap with later cohort reports.
Liu JJ et al. (2025) [40]	Prospective T2D cohorts; discovery n = 1,826, validation n = 1235; median follow-up 9.2/4.0 y.	Targeted organic-acid measurement. Baseline urine mass spectrometry.	Lactate, pyruvate, citrate, α-ketoglutarate, succinate, fumarate and malate.	Composite renal-outcome HRs per SD: fumarate 1.82 (95% CI 1.17–2.82), citrate 0.83 (0.72–0.96); both remained associated after mutual metabolite adjustment.	Cardiorenal and mutual metabolite adjustment; independent-cohort replication of metabolite–renal outcome associations.	Composite includes doubling of creatinine, not ESKD alone; T2D-specific; no test-guided intervention.
Martinez PJ et al. (2020) [41]	Cross-sectional age-stratified study; 234 adults; age groups 30–50, 50–70 and > 70 y.	Broader urinary metabolomics. Spot urine; NMR discovery and targeted MS; total spectral-intensity normalization for NMR.	2-Hydroxybutyrate, oxaloacetate, citrate, glucuronate and pantothenate.	Age-dependent profiles distinguished current cardiovascular risk/prior events; glucuronate and TMAO spanned age groups.	Age/risk-group analyses; targeted-MS analytical confirmation; internal Monte Carlo cross-validation using repeated two-thirds/one-third splits.	Cross-sectional classification, not incident-event prediction; small subgroups and renal differences.
Hertel J et al. (2022) [17]	Population multi-omics association study; 951 SHIP adults.	Broader urinary metabolomics. Spontaneous urine stored at −80°C; urine NMR and stool 16 S sequencing; creatinine normalization and spectral total-area scaling.	Hippurate, succinate, formate and 4-hydroxyphenylacetate; related microbial co-metabolites.	Microbial diversity/taxa associated with urinary metabolites; predicted hippurate statistically mediated health associations.	Demographic, metabolic, renal, lifestyle, and batch adjustment; FDR and bootstrap analyses; internal 10-fold cross-validation of microbial prediction models.	Cross-sectional mediation is not causal evidence; no stand-alone microbiome diagnostic validation; SHIP overlap.
Li T et al. (2024) [42]	Multi-cohort study; reference sample n = 994; clinical analyses up to n = 5989.	Broader urinary metabolomics. Morning spot urine; quantitative NMR; creatinine normalization.	61 reference metabolites; 12 automated markers include citrate, hippurate and 2-hydroxyisobutyrate.	362 clinical/biochemical associations for 12 markers; insulin adjustment affected many BMI associations.	Sex/cohort-specific age adjustment; additional clinical-covariate analyses; three cohorts and analytical-variation assessment.	Cross-sectional; 61 reference metabolites differ from the 12 clinical markers; preventive utility untested.
Valo E et al. (2025) [16]	Three-cohort GWAS meta-analysis/MR; 8011 participants, including general-population and T1D cohorts.	Broader urinary metabolomics. Spot urine; cohort-specific fasting procedures; −80°C storage; NMR and creatinine normalization.	54 urinary metabolite traits; broad metabolomic profile.	54 genetic associations involving 19 metabolites; MR suggested eGFR effects on 13 urinary metabolites.	Three-cohort genetic association meta-analysis and two-sample Mendelian randomization; no clinical prediction-model evaluation.	European-ancestry/T1D context; MR assumptions; renal effects do not establish clinical utility.
Gauβ KF et al. (2026) [43]	Age-model development/validation; SHIP-TREND n = 4184 (20–84 y), SHIP-START n = 3701 (20–81 y); repeated samples n = 2160/1441.	Broader urinary metabolomics. Urine stored at −80°C; 600-MHz ^1^H NMR; creatinine normalization with PQN sensitivity analysis.	77 retained urinary metabolites; broad profile.	External age MAE 11.65 y (R^2^ = 0.38); mortality HRs per SD age acceleration 1.23 (95% CI 1.12–1.36) and 1.13 (1.06–1.21).	Age, sex, BMI, smoking, diabetes and activity adjustment; internal 10-fold cross-validation in SHIP-TREND; external evaluation of the trained age model in SHIP-START; longitudinal repeated measurements.	External cohort from same region; SHIP overlap; broad score does not validate a conventional UOA panel.
Hertel J et al. (2016) [44]	Urinary age-score development; two population samples with ∼13-y survival follow-up; bariatric-surgery evaluation.	Broader urinary metabolomics. Urinary ^1^H NMR.	Broad urinary metabolomic age score.	Age score associated with aging phenotypes and survival; also evaluated after bariatric surgery.	Age-score associations assessed in two independent population samples, survival follow-up and bariatric-surgery assessment.	Individual-acid effects not established here; potential overlap with other SHIP reports; clinical utility unproven.
Kobelyatskaya AA et al. (2025) [45]	Neural-network age-model development; 863 urine samples (not necessarily unique participants).	Targeted organic-acid measurement. Urinary organic-acid assay.	60-acid model and reduced 10-marker model.	Organic-acid-based chronological-age prediction models developed.	–	Age prediction alone does not establish health benefit.
Teruya T et al. (2020) [25]	Cross-sectional comparison; 27 healthy adults: 13 aged 25–38 y and 14 aged 69–81 y.	Broader urinary metabolomics. First-morning midstream after ≥8-h fast; HILIC–Orbitrap LC-MS; peak-area analysis with separate creatinine-normalized comparisons.	2-/3-Hydroxybutyrate, citrate, malate, hippurate and quinolinate within 99 metabolites.	55/99 metabolites differed by age (53 lower, 2 higher in older adults); hippurate was not age-associated.	Group comparisons/correlations; repeat sampling in 3 young adults over 5 days; no independent cohort.	n = 27; cross-sectional; creatinine normalization altered age associations; no prospective validation.
Nik Mohd Fakhruddin NNI et al. (2020) [46]	Cross-sectional pilot; 27 older Malaysians: 9 successful aging, 9 usual aging and 9 MCI.	Broader urinary metabolomics. Second-void urine stored at −80°C; ^1^H NMR; total-area normalization, log transformation and unit-variance scaling.	Citrate and succinate within a broader profile.	Citrate and several other metabolites were higher in the successful-aging group.	Demographic/lifestyle covariates in dietary analyses; PLS-DA permutation and CV-ANOVA; no independent cohort.	Only 9 participants/group; dietary sensitivity and overfitting risk; no longitudinal aging outcome.
Chen L et al. (2020) [47]	Cross-sectional pilot; 33 older and 33 young healthy Singaporean men.	Broader urinary metabolomics. NMR profiling of urine and serum.	Urinary citrate, ascorbate and acetate.	Older men had higher urinary citrate/ascorbate and lower acetate.	–	Small, male-only sample; cross-sectional; serum findings cannot be attributed to urine.
Swann JR et al. (2013) [48]	Two-cohort cross-sectional study; Taiwan SEBAS n = 857 (54–91 y), US MIDUS II n = 1148 (35–86 y).	Broader urinary metabolomics. 12-h overnight urine; ^1^H NMR with spectral-integral normalization; UPLC-MS confirmation.	β-Hydroxy-β-methylbutyrate; related co-metabolites PAG and p-cresyl sulfate.	Hydroxy-methylbutyrate/creatine decreased with age in both cohorts; microbial co-metabolites increased.	Age/sex analyses; association replication across two populations; internal 7-fold cross-validation and permutations testing; selected UPLC-MS analytical confirmation.	Cross-sectional age gradients; dietary/cultural differences; excretion is not a direct measure of microbial function.
Collino S et al. (2013) [49]	Cross-sectional multi-matrix study; overall cohort: 143 centenarians, 210 offspring, 73 age-matched controls and 21 young adults; 21–111 y.	Broader urinary metabolomics. First-morning urine stored at −80°C; urine NMR alongside serum MS.	Urinary PAG and p-cresyl sulfate; related co-metabolites.	Urinary PAG and p-cresyl sulfate increased in centenarians.	Multivariate age-group comparisons; OPLS-DA discrimination and microbiome–metabolite association analyses.	Survivor selection/sex imbalance; overall counts are not urine-analysis counts; serum lipid findings are separate.
Moaddel R et al. (2024) [50]	Cross-sectional GESTALT study; ages 22–92 y; urine n = 82 (plasma n = 101; muscle n = 88).	Broader urinary metabolomics. ≥8-h fasting urine stored at −80°C; targeted metabolomics with internal QC; no creatinine normalization.	Broad targeted metabolite panel.	35 urinary metabolites associated with age; patterns differed across compartments.	Sex, race, and BMI adjustment; cystatin-C sensitivity analysis; independent-cohort assessment concerned plasma findings, not urinary findings.	Small, selected healthy cohort; shared GESTALT data with 2025 report; pathways do not directly measure mitochondrial function.
Lin Y et al. (2026) [51]	Cross-sectional TwinsUK study; 1195 women; mean age 58.96 ± 14.59 y.	Broader urinary metabolomics. Paired urinary metabolomics and stool shotgun metagenomics; urinary measurements by Metabolon.	Six-metabolite panel including cinnamoylglycine; broader urinary metabolomic profile.	Six-metabolite panel distinguished low from high Shannon diversity; test-set AUROC 0.732.	Age, BMI, batch, and family structure adjustment; grouped 80%/20% training–test split within TwinsUK; five-fold cross-validation during model development.	Female twin cohort; cross-sectional; same-cohort testing; no external validation or demonstrated clinical utility.
Moaddel R et al. (2025) [52]	GESTALT multi-omics age modelling; 22–92 y; urine proteomics n = 95 baseline/65 follow-up; earlier urine metabolomics n = 82.	Broader urinary metabolomics. ≥8-h fasting urine; proteomics plus prior metabolomics; Olink NPX processing applies to proteins.	Broad metabolite and protein profiles; not an organic-acid-specific model.	Compartment-specific age scores associated with aging phenotypes.	Sex, race, and BMI adjustment; renal sensitivity analyses; repeated internal five-fold cross-validation and permutation testing; Independent-cohort assessment concerned plasma signatures.	Shared GESTALT data; modality-specific counts are not combined-model counts; no validated test-guided intervention.
Robinson O et al. (2020) [53]	Age-model development; Airwave n = 2239; Finnish validation subset n = 2144.	Broader urinary metabolomics. NMR and LC-MS of urine plus serum; comparison with DNA methylation.	Untargeted urine/serum features; mixed-matrix score.	Age-model correlation ∼0.86 across test sets; age acceleration associated with obesity and other risk factors.	Clinical and lifestyle adjustment; repeated 80%/20% training–test splits and internal cross-validation; Finnish follow-up assessed selected serum metabolite age associations.	Mixed matrices; subset validation does not validate a complete urine-only or conventional UOA model.
Kneipp A et al. (2026) [54]	Cross-sectional MEIA study; digit span n = 560, verbal fluency n = 553; recruitment ages 18–75 y.	Broader urinary metabolomics. Morning spot urine after ≥12-h fast; ^1^H NMR; creatinine-normalized ratios ×100.	51 metabolites including hydroxyhippurate, trans-aconitate, pyroglutamate, acetate, citrate and hippurate.	No cognitive associations survived multiple-testing correction.	Age, sex, BMI, education, lifestyle and cognition-related conditions; multiplicity correction; no independent cohort.	Cross-sectional; corrected null findings do not support established cognitive prediction.
Rabassa M et al. (2020) [55]	Prospective InCHIANTI study; 119 Italians aged ≥65 y; 3-y follow-up.	Broader urinary metabolomics. Baseline 24-h urine; HPLC-QToF-MS.	Nut-exposure polyphenol/fatty-acid metabolites; broad dietary signature.	Cognitive-decline OR 0.995 (95% CI 0.991–0.999) per marker-model unit; AUROC 0.731 (0.625–0.837).	Baseline cognition, demographic, lifestyle and clinical adjustment; same-cohort prospective assessment; no external validation.	Small observational sample; model-unit OR is not a per-SD effect; residual dietary/lifestyle confounding.
Bédard-Delisle C et al. (2026) [56]	Uncontrolled pre–post pilot; 53 community-dwelling adults aged ≥70 y; 3 months.	Targeted organic-acid measurement. Four monthly fasting, filter-paper urine samples; UPLC-MS/MS; MMA/creatinine ratio.	Methylmalonic acid.	MMA/creatinine decreased after oral B12 (1200 μg/day); balance/cognition improved; MMA change correlated with cognitive change.	Within-person comparisons/correlations; no randomized control.	Uncontrolled; practice effects/regression to the mean; does not validate broad UOA-directed supplementation.
Ruan Y et al. (2019) [57]	Double-blind randomized trial; 59 adults with T2D; fish/flaxseed/corn oils for 180 days.	Broader urinary metabolomics. Morning urine before/after intervention; UHPLC-Orbitrap MS.	CMPF and dihydrojasmonic acid; carnitines in the broader panel.	Fish oil increased urinary CMPF versus corn oil (p = 0.004); urine/serum changes correlated (r = 0.646, p < 0.001).	Randomized-group comparisons/correlations; no external biomarker validation.	Dietary exposure response does not demonstrate benefit from UOA-guided care.
Tsoukalas D et al. (2017) [58]	Reference-distribution study; 122 healthy adults; 21–81 y, mean 42.0 ± 9.6 y.	Targeted organic-acid measurement. Early-morning urine stored at −80°C; targeted GC-MS.	22 acids, including citrate, isocitrate, malate, succinate, α-ketoglutarate, pyruvate, lactate, MMA and pyroglutamate.	Healthy-adult percentile distributions reported; no significant sex differences.	Descriptive/sex-group analyses; analytical assay and reference distributions; no disease-based external validation.	Single reference sample; confidence intervals of means are not clinical reference intervals; no diagnostic thresholds established.
Thévenot EA et al. (2015) [59]	Cross-sectional metabolomics study; 183 adults.	Broader urinary metabolomics. LC-HRMS with univariate and OPLS analyses.	108 characterized metabolites; broad urinary profile.	Metabolite variation associated with age, BMI and sex.	Age/BMI/sex analyses; univariate testing and OPLS workflow.	Physiological variation, not clinical utility; dataset reused by Wang et al. (2021).
Wang T et al. (2021) [60]	Reanalysis of the same 183-person dataset as Thévenot et al. (2015).	Broader urinary metabolomics. Reused LC-HRMS data; 109 annotated compounds.	p-Anisic acid, 3-methylcrotonylglycine and N-acetylaspartate among the broader profile.	30 age-, 18 BMI- and 42 sex-related metabolites reported.	Age/BMI/sex comparisons and correlations; reanalysis, not independent replication.	Same participants as Thévenot et al. (2015); not a new independent cohort.
Boutin M et al. (2020) [61]	Analytical method comparison; paired samples from 34 older NuAge participants.	Targeted organic-acid measurement. Urine UPLC-MS/MS versus GC-MS; MMA/creatinine ratio and paired serum MMA.	MMA, chromatographically separated from succinate.	Intra-/interday accuracy bias and precision coefficients ≤6.3%; strong method correlations.	Analytical performance assessment and paired UPLC-MS/MS–GC-MS comparison; no clinical prediction-model evaluation.	Analytical performance does not establish screening accuracy, clinical thresholds or treatment benefit.
Hallan S et al. (2017) [62]	CKD case–control study with replication/drug substudy; discovery 22 cases/10 controls, replication 45/15; adult eligibility ≥18 y.	Targeted organic-acid measurement. ∼24-h urine collected on ice, stored at −80°C; GC-MS; creatinine-standardized aliquots converted to 24-h excretion.	Citrate, cis-aconitate, isocitrate, 2-oxoglutarate and succinate.	TCA metabolite excretion 40–68% lower in CKD; citrate increased 74% during paricalcitol treatment (p = 0.00009).	Replication controls matched for age, sex, and race; independent-cohort replication of urinary metabolite differences, complementary renal gene expression and drug-response analyses.	Small, disease-specific groups; excretion does not directly quantify mitochondrial respiration; preventive utility untested.
Hall AM et al. (2015) [63]	Cross-sectional mitochondrial-disease cohort; 117 adults, including 75 m.3243 A > G carriers; omics performed in a subset.	Broader urinary metabolomics. Urine NMR metabolomics, MS proteomics and RBP/albumin assays.	Broad metabolomic profile; RBP/albumin are protein markers.	RBP elevated in 29/75 carriers and albumin in 23/75; subset omics profiles differed, including in asymptomatic carriers.	Disease/control and diabetes/clinical-phenotype comparisons.	117 is not the metabolomics sample size; inherited disease and renal involvement limit preventive extrapolation.
Andrade-Vieira R et al. (2026) [64]	Cross-sectional discovery/clinical evaluation; biobank 200 serum/80 urine samples; additional urine cohort n = 60 (30 men/30 women), 50–70 y.	Broader urinary metabolomics. Clinical cohort: first-morning urine after ≥8-h fast, −80°C; urine colorimetric/fluorometric assays; serum LC-MS.	Succinate plus glutamate, xanthine, taurine and carnitine.	Combined panel associated with muscle-related phenotype; individual metabolites were insufficient.	Combined logistic models; comparison with DXA, SPPB, grip strength and IPAQ.	Cross-sectional classification, not future sarcopenia prediction; small clinical cohort; different serum/urine platforms.
Rawal PM et al. (2025) [65]	Cross-sectional renal acid-balance study; 245 participants (190 stone formers, 55 controls); 19–92 y, mean 55 y.	Aggregate organic anion measurement. 24-h urine; supplements stopped 1 week; titration across pKa ranges; creatinine normalization and 24-h excretion.	Aggregate organic anions by pKa; routine chemistry including citrate.	pKa 2.70–3.60 fraction increased with age and associated with ammonia (both p < 0.001).	Age/sex, stone status and acid-balance models; creatinine as applicable; triplicate titrations.	Mainly stone formers; few older controls; bulk titration cannot resolve specific acids or establish mitochondrial dysfunction.

Abbreviations. ^1^H NMR, proton nuclear magnetic resonance spectroscopy; ALT, alanine aminotransferase; AUROC, area under the receiver operating characteristic curve; BMI, body mass index; CI, confidence interval; CKD, chronic kidney disease; CMPF, 3-carboxy-4-methyl-5-propyl-2-furanpropanoic acid; CV-ANOVA, analysis of variance of cross-validated residuals; DNA, deoxyribonucleic acid; DXA, dual-energy X-ray absorptiometry; eGFR, estimated glomerular filtration rate; ESKD, end-stage kidney disease; FDR, false discovery rate; FIB-4, fibrosis-4 index; FIGLU, formiminoglutamate; GC-MS, gas chromatography–mass spectrometry; GWAS, genome-wide association study; HILIC, hydrophilic interaction liquid chromatography; HOMA2-IR, updated homeostasis model assessment of insulin resistance; HPLC, high-performance liquid chromatography; HR, hazard ratio; IPAQ, International Physical Activity Questionnaire; LC-HRMS, liquid chromatography–high-resolution mass spectrometry; LC-MS, liquid chromatography–mass spectrometry; MAE, mean absolute error; MCI, mild cognitive impairment; MetS, metabolic syndrome; MMA, methylmalonic acid; MR, Mendelian randomization; MRI, magnetic resonance imaging; MS, mass spectrometry; MS/MS, tandem mass spectrometry; NAFLD, nonalcoholic fatty liver disease; NMR, nuclear magnetic resonance; NPX, normalized protein expression; OPLS, orthogonal partial least squares; OR, odds ratio; PAG, phenylacetylglutamine; PLS-DA, partial least squares discriminant analysis; OPLS-DA, orthogonal partial least squares discriminant analysis; PQN, probabilistic quotient normalization; QC, quality control; QToF, quadrupole time-of-flight; RBP, retinol-binding protein; ROC, receiver operating characteristic; SD, standard deviation; SPPB, Short Physical Performance Battery; T1D/T2D, type 1/type 2 diabetes; TCA, tricarboxylic acid; TMAO, trimethylamine N-oxide; UPLC, ultra-performance liquid chromatography; UHPLC, ultra-high-performance liquid chromatography; UOA, urinary organic acid; UV, ultraviolet; y, years; 4-HPPA, 4-hydroxyphenylpyruvic acid. Named cohort abbreviations follow the cited reports.

Of the 38 human-study reports, nine evaluated targeted urinary organic acids or organic-acid-focused panels [8], [37], [39], [40], [45], [56], [58], [61], [62], 28 investigated broader urinary metabolomic profiles or mixed-metabolite panels [16], [17], [25], [32], [33], [34], [35], [36], [38], [41], [42], [43], [44], [46], [47], [48], [49], [50], [51], [52], [53], [54], [55], [57], [59], [60], [63], [64], and one assessed aggregate organic anion excretion by titration [65]. Six reports included cross-omics statistical analyses or comparisons of omics-derived scores [16], [17], [49], [51], [52], [53], while two studies provided complementary parallel or pathway-level omics evidence [62], [63]. These multi-omics classifications overlap with the urinary measurement categories and do not imply development or external validation of an integrated prediction model.

### 4.1 Established diagnostic applications and foundational studies

UOA analysis is established in the diagnostic evaluation of inherited metabolic disorders [7], [66]. However, findings from disorders characterized by specific enzymatic defects and pronounced metabolic abnormalities cannot be directly extrapolated to preventive screening or healthy-aging assessment. A study of 122 healthy adults aged 21–81 years characterized distributions of 22 urinary organic acids using GC-MS [58]. Similarly, method comparison of urinary methylmalonic acid/creatinine in 34 older adults established analytical performance rather than the clinical effectiveness of broad UOA screening [61].

### 4.2 Human studies in metabolic and nutritional contexts

Cross-sectional studies support associations with cardiometabolic phenotypes. In 529 Korean adults, high levels of selected organic acids were associated with metabolic syndrome, with age- and sex-adjusted odds ratios of 1.915–2.809 [8]; associations for pyruvate, formiminoglutamate, and quinolinate were not independent of insulin resistance [8]. In 11,754 European participants, a broader 17-metabolite signature classified prevalent metabolic syndrome with area under the receiver operating characteristic curve (AUROC) values of 0.83–0.87 [32]. More extensive replication is available for some population associations. A study of 1880 US participants identified 29 urinary metabolites associated with body mass index (BMI), of which 25 replicated in 444 UK participants, with repeat 24-h collections supporting reproducibility [34]. However, BMI associations do not establish clinical decision thresholds. In 133 Korean adults evaluated by magnetic resonance imaging (MRI) and elastography, several steatosis associations attenuated after adjustment for BMI and insulin resistance, illustrating the importance of confounding [37].

Prospective evidence is available in defined disease populations. Urinary TCA-related metabolites were evaluated in discovery and independent replication cohorts of 1826 and 1235 people with type 2 diabetes [40]. Higher fumarate and lower citrate were associated with a composite renal outcome: adjusted hazard ratios per SD were 1.82 (95% CI 1.17–2.82) and 0.83 (0.72–0.96), respectively, before additional mutual metabolite adjustment; both remained associated after that adjustment [40]. The composite included sustained severe renal impairment, dialysis, renal death, or doubling of serum creatinine, extending beyond end-stage kidney disease alone [40]. These findings support disease-specific prognostic associations.

Nutritional studies also require endpoint-specific interpretation. A randomized oil-supplementation study in 59 adults with type 2 diabetes demonstrated a urinary 3-carboxy-4-methyl-5-propyl-2-furanpropanoic acid (CMPF) response to fish oil, supporting its role as an exposure marker [57]. An uncontrolled vitamin-B12 pilot reported reductions in urinary methylmalonic acid (MMA)/creatinine and improvements in clinical measures [56]. However, its design cannot establish treatment causality or justify extrapolation to broad UOA-directed supplementation.

### 4.3 Mitochondrial function and energy metabolism

Human mechanistic studies provide disease-specific support for altered urinary energy-metabolism profiles. A comparison of 22 adults with non-diabetic chronic kidney disease (CKD) and 10 controls, with replication in 45 CKD patients and 15 controls, found 40–68% reductions in urinary excretion of several TCA-cycle metabolites [62]. Accompanying renal gene-expression findings were consistent with altered metabolic pathways [62]. Nevertheless, urinary excretion is influenced by renal transport and systemic metabolism and is not a direct measurement of mitochondrial respiratory capacity. A separate study identified frequent tubular or glomerular abnormalities in a cohort of 117 adults with mitochondrial disease and altered urinary omics profiles in a subset [63]. Together, these studies support mechanistic investigation but do not establish that a urinary organic acid pattern can diagnose nonspecific mitochondrial dysfunction in otherwise healthy adults.

### 4.4 Host–microbiome interactions

In 951 participants from the Study of Health in Pomerania (SHIP), paired stool 16 S rRNA gene sequencing and urinary NMR measurements revealed associations between gut microbial features and urinary hippurate, succinate, formate, and 4-hydroxyphenylacetate [17]. Models incorporated demographic, metabolic, renal, and lifestyle covariates, and additional analyses examined microbial prediction and statistical mediation of health associations [17]. These analyses support hypotheses about host–microbial metabolism, but cross-sectional statistical mediation does not demonstrate causality or validate urine profiles as stand-alone measures of microbiome composition. Renal handling further limits simple biological attribution. A three-cohort genetic study of 8011 participants found genetic associations for urinary metabolites and Mendelian-randomization evidence suggesting that eGFR influences 13 urinary metabolite concentrations [16].

### 4.5 Aging-related associations and outcomes

The aging-related evidence summarized here includes cross-sectional age differences, metabolomic age models, and associations with survival, cognition, or physical function. These endpoints address different questions and should not be treated as interchangeable evidence of healthy aging. Evidence from metabolic syndrome, inherited metabolic disorders, or other disease-specific populations does not independently establish preservation of function or benefit from UOA-guided care in older adults.

Age-related urinary differences have been observed, but their interpretation depends on sampling, normalization, and study design. A study of 27 adults identified 55 age-associated metabolites; many correlated with creatinine, whereas hippurate was not age-associated [25]. An investigation of two populations totaling 2005 participants identified common age gradients using overnight urine collections and cross-platform confirmation for selected co-metabolites [48]. Urinary metabolomic age scores have also been investigated in relation to survival [44]. More recently, an age-prediction model was developed in 4184 SHIP-TREND participants and evaluated in 3701 SHIP-START participants [43]. External age-prediction error was 11.65 years (R^2^ = 0.38), and adjusted mortality hazard ratios per SD of the age-independent score were 1.23 (95% CI 1.12–1.36) and 1.13 (1.06–1.21), respectively [43]. These results support prognostic association of a broad urinary score, not clinical validation of a conventional UOA panel. SHIP-based reports also require attention to cohort overlap. AcidAGE more directly addresses organic-acid-based age estimation through models using urinary organic acid measurements [45]. However, estimation of chronological age alone may not establish clinical utility for preventive care.

Null and exploratory findings are equally relevant. In the Metabolism, Nutrition and Immune System in Augsburg (MEIA) study, associations between urinary metabolites and cognitive performance (assessed using digit span in 560 participants and verbal fluency in 553) were no longer statistically significant after correction for multiple testing [54]. A newer sarcopenia study evaluated a urinary panel including succinate against muscle and physical-performance measures using a cross-sectional design [64]. Evidence therefore supports further assessment of selected biomarkers and composite scores, while the clinical benefit of UOA-guided preventive or healthy-aging care remains unestablished in the studies summarized here.

## 5 Clinical interpretation and evidentiary boundaries

### 5.1 Measurement and interpretation according to intended use

UOA measurements can characterize differences in metabolite concentrations or excretion and support investigation of associations with clinical and physiological variables [42]. Repeated sampling can characterize within-person variation, although urinary profiles are affected by sampling conditions and biological variability [30]. These measurements should not be interpreted as direct evidence of impaired enzymatic efficiency, micronutrient insufficiency, or reduced metabolic resilience without independent validation of the proposed interpretation.

Interpretation depends on the assay and clinical question. Organic acid testing has established diagnostic applications in inherited metabolic disorders [4]. In preventive research and commercial functional assessment, metabolite abnormalities or pathway associations require separate validation before being used for diagnosis, prognosis, or treatment selection. Metabolite pattern recognition and biological interpretation are distinct analytical steps [31]. Pathway interpretation also depends on the reliability of metabolite identification and functional annotation [67]. Such interpretation, however, does not itself establish a validated clinical decision threshold.

### 5.2 Clinical context and interpretative boundaries

Limitations of exploratory urinary profiles and commercial preventive panels should not be generalized to validated disease-specific applications. Such profiles should not be used to infer organ-specific pathology or guide treatment without evidence supporting the particular assay and interpretation.

Interpretation of urinary organic acids in older adults should incorporate contemporaneous kidney-function measurements, age, sex, muscle mass or sarcopenia status, hydration and collection conditions, and medication exposure. Where low muscle mass may compromise creatinine-based eGFR, cystatin C–based assessment can provide complementary information [20]. Research analyses should evaluate the influence of renal function and normalization strategy through appropriate adjustment, stratification, or sensitivity analyses. Neither an elevated metabolite-to-creatinine ratio nor a longitudinal change should automatically be interpreted as increased metabolite production or deterioration in systemic metabolic function.

The physiological and methodological determinants discussed in Section 3 should therefore inform interpretation of individual results. Analytical performance and exploratory associations should be distinguished from evidence supporting clinical implementation; diagnostic, prognostic, and treatment claims require validation for the specified assay and intended use [12].

## 6 Potential applications in preventive health assessment and healthy aging

### 6.1 Aging associations and longitudinal research

Urinary metabolomics has been investigated through cross-sectional comparisons of age groups [25] and longitudinal analyses of metabolomic age scores [43]. These findings support further aging research, but do not establish the clinical effectiveness of UOA-guided preventive assessment. Cross-sectional differences between age groups should be distinguished from within-person changes over time; longitudinal interpretation requires repeated measurements.

Human urinary metabolomics studies have identified age-associated differences in metabolites related to several biochemical pathways [25]. Such differences describe associations with age and do not, by themselves, demonstrate preclinical impairment, reduced metabolic flexibility, or an actionable nutrient deficiency. Study-level findings and their limitations are summarized in Table 3.

### 6.2 Relevance to preventive and healthy-aging applications

Urinary metabolomics studies in older adults suggest that urinary metabolic profiles differ between aging phenotypes and may reflect dietary patterns and functional aging status [46]. These cross-sectional findings support associations with dietary patterns, but do not demonstrate responsiveness to lifestyle interventions [46]. For healthy-aging applications, the relevant question is whether these measurements inform preservation of function or prevention of clinically meaningful decline.

Prospective associations identified using particular urinary metabolites or metabolomic models support further biomarker research, but cannot be transferred automatically to commercial preventive panels. Clinical adoption requires validation of the specified assay, population, decision rule, and intended use, including evidence that test-guided management improves relevant outcomes.

## 7 Integration into multi-omics approaches

UOA measurements may complement genomic, proteomic, and microbiome data, but theoretical integration, exploratory human studies, and externally validated predictive models represent distinct levels of evidence. Combining molecular datasets can support biological interpretation; it does not itself establish diagnostic or prognostic utility. Fig. 2 summarizes findings from selected studies in Table 3, illustrating how urinary metabolomic measurements have been investigated alongside microbial, proteomic, and genomic information and interpreted within clinical context.

**Fig. 2:**
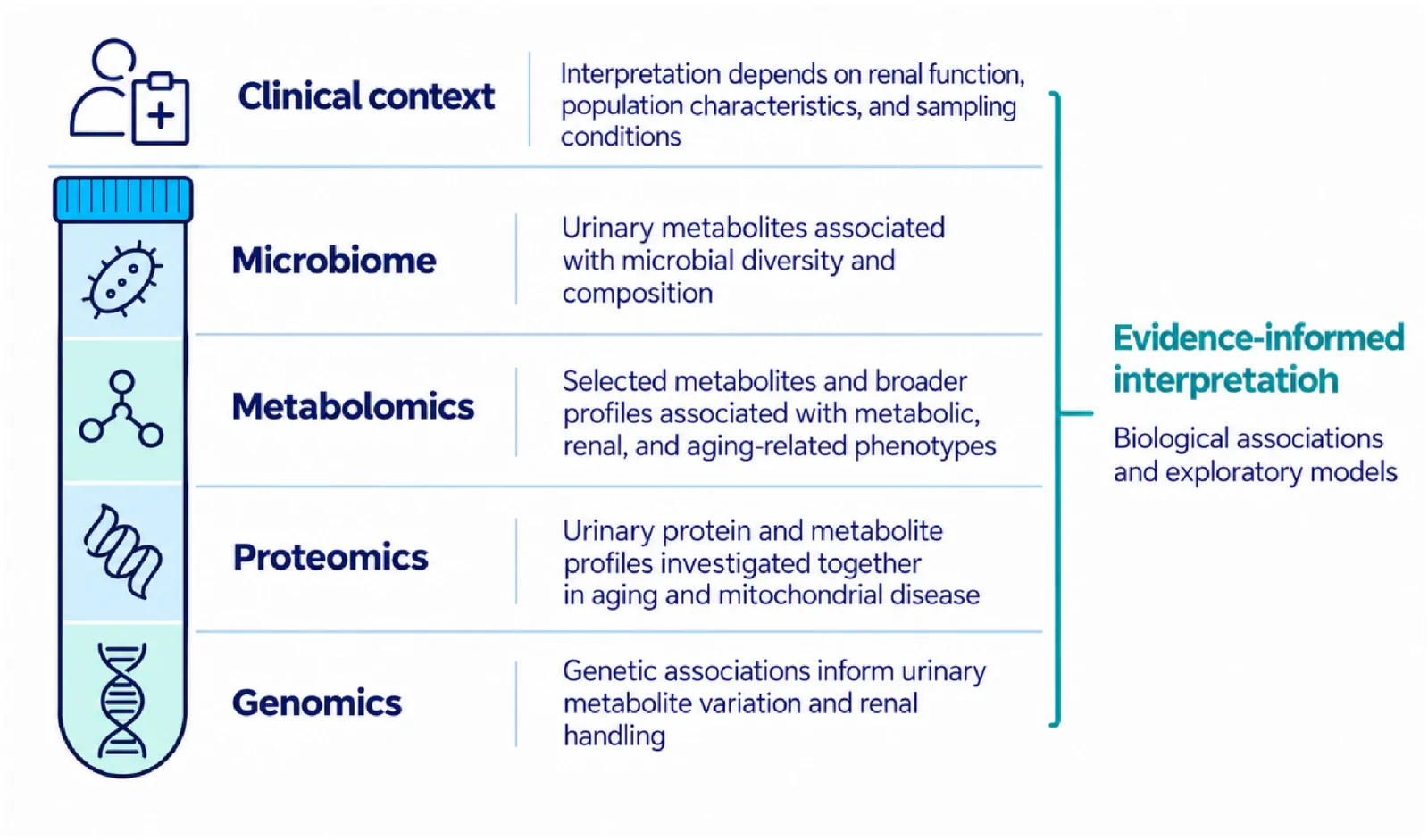
Evidence-informed framework for urinary metabolomics and multi-omics integration in preventive health assessment. The figure summarizes selected investigations included in Table 3. Clinical interpretation requires consideration of renal function, population characteristics, and sampling conditions [16], [42], [65]. Microbiome studies link urinary metabolites with microbial diversity and composition [17], [51]. Metabolomic investigations report associations with metabolic phenotypes, renal outcomes, and aging-related characteristics [8], [32], [34], [39], [40], [43]. Complementary urinary proteomic and metabolomic investigations address aging and mitochondrial disease [52], [63], while genetic analyses inform urinary metabolite variation and renal handling [16]. The molecular layers represent separate study-specific investigations, not a single externally validated multi-omics model. The segmented tube is a schematic organizing device and does not imply that all molecular measurements were obtained from urine.

### 7.1 Theoretical integration

Genetic, microbiome, and renal gene-expression studies illustrate how urinary organic acids can be investigated alongside other molecular measurements to explore the determinants of metabolite excretion [16], [17], [62]. Such integration supports biological hypotheses, but associations or concordant pathway findings should not be interpreted as direct evidence of causality or clinical utility. Renal transport and kidney function also influence urinary metabolite concentrations, limiting their interpretation as direct measures of intracellular metabolic flux or enzymatic efficiency [16]. Evidence should therefore remain specific to the metabolites, analytical methods, populations, and models investigated; it should not automatically be extrapolated to a different targeted assay or commercial panel.

### 7.2 Human evidence involving urinary organic acids

A study of 951 participants combined urinary NMR metabolomics with stool 16 S rRNA sequencing and identified associations between microbial features and urinary organic acids, including hippurate, succinate, formate, and 4-hydroxyphenylacetate [17]. An internally cross-validated model using microbial genus abundances explained 12.7% of the variance in urinary hippurate; its prediction score was also associated with inflammatory, lipid, and liver-related measures [17]. These findings provide exploratory evidence linking microbial composition with urinary organic acid variation, but do not establish causal mediation or an externally validated predictor of healthy-aging outcomes.

A subsequent study combined urinary metabolomics with stool shotgun metagenomics in 1195 women. A six-metabolite panel, including cinnamoylglycine, distinguished low from high Shannon diversity with an AUROC of 0.732 in a held-out test set [51]. However, testing involved participants from the same cohort, and the panel included metabolites beyond organic acids; this represents proof-of-concept prediction of microbial diversity rather than external validation or demonstrated clinical utility [51].

Genetic integration has also been examined in a genome-wide association meta-analysis of 54 urinary NMR metabolites across three cohorts comprising 8011 participants [16]. Among the findings, a variant in SLC13A2, which encodes a renal citrate transporter, was associated with urinary citrate concentration [16]. This illustrates how genetic data can inform the interpretation of urinary organic acid variation, particularly through renal handling. However, a multi-cohort association analysis is distinct from external validation of a clinical prediction model.

Evidence linking urinary organic acids with tissue-level molecular measurements is available from non-diabetic chronic kidney disease. Urinary citrate, cis-aconitate, isocitrate, 2-oxoglutarate, and succinate excretion was 40–68% lower in affected participants, with reductions replicated in an independent cohort; separate renal biopsy analyses showed concordant reductions in expression of genes associated with the TCA cycle [62]. This provides disease-specific biological support, but the measurements were not combined into an externally validated prediction model and should not be extrapolated to healthy-aging assessment.

### 7.3 External validation and clinical relevance

The examples above support biological investigation rather than established preventive or healthy-aging applications. External validation of a multi-omics prediction model requires evaluation of a prespecified model in an independent population for a defined outcome, including assessment of discrimination and calibration [68]. Internal cross-validation, replication of individual associations, or external validation of a metabolomics-only model should not be described as external validation of an integrated multi-omics model. External validation has been reported for a broader urinary NMR age model developed in 4184 participants and evaluated in an independent cohort of 3701 participants [43]. This illustrates external validation of a urinary metabolomics model, but does not establish external validation of a multi-omics model or an organic-acid-specific assay [43].

Future studies should establish whether adding urinary organic acids improves prediction beyond conventional clinical variables and the other molecular layers, rather than relying on statistically significant associations alone [69]. For proposed applications in aging research, this requires longitudinal evaluation against meaningful outcomes, such as incident frailty or functional decline, with appropriate consideration of renal function and other determinants of urinary excretion. Predictive performance should also be distinguished from clinical impact: prospective comparative studies are needed to determine whether using a model improves clinical decisions and patient outcomes [70].

## 8 Evidence limitations and research priorities

### 8.1 Methodological and interpretative limitations

Biological and pre-analytical variability limits comparability across studies. Standardization can reduce avoidable variation, but does not eliminate biological differences or establish equivalence between assays. Outside validated disease-specific applications, changes in urinary organic acids may have multiple biological explanations. Their specificity for a proposed preventive diagnosis must therefore be demonstrated for the particular assay, population, and decision rule. Clinical translation additionally requires reproducible analytical procedures and independent validation of candidate biomarkers [12].

Describing urinary metabolite patterns as ‘functional’ does not establish that they identify a clinically meaningful impairment. In preventive applications, interpretations concerning metabolic vulnerability, resilience, or micronutrient insufficiency require explicit definitions, reproducible measurement criteria, and validation against relevant outcomes. Where such evidence is unavailable, findings should be described as metabolite differences or associations rather than validated clinical states.

Regarding the review methodology, restriction to PubMed may have resulted in omission of relevant studies indexed elsewhere. Nevertheless, eight complementary, topic-specific search strategies and targeted reference-list checking supported coverage of the review's principal themes.

### 8.2 Limitations of outcome validation

The human evidence summarized in Section 4 includes cross-sectional, exploratory, and prospective investigations, which support different levels of inference. Cross-sectional studies alone cannot establish prediction of future outcomes. However, prospective associations have been reported between selected urinary metabolites and renal outcomes in people with type 2 diabetes [40]. Broader urinary metabolomic age scores have also shown prospective associations with mortality [43].

These findings remain specific to the investigated populations, assays, and outcomes; they do not establish the clinical benefit of commercial preventive panels or UOA-guided care. Similarly, replication of an individual metabolite association or external validation of a metabolomics-only model should not be interpreted as external validation of an integrated multi-omics model.

### 8.3 Research priorities

Future studies should prioritize longitudinal cohorts with repeated measurements, standardized collection and analytical procedures, and clearly defined preventive or aging-related outcomes. Relevant endpoints include incident frailty, functional decline, and other outcomes that reflect health rather than chronological age alone. Study design and analysis should account for renal function, muscle mass, medication exposure, and the chosen normalization strategy.

For predictive and multi-omics applications, the requirements should guide evaluation of independent validation and added value beyond conventional clinical measures. Prospective comparative studies are also needed to determine whether using urinary organic acid measurements improves clinical decisions or patient outcomes. These priorities address the remaining gap between biological association and demonstrated clinical utility.

## 9 Conclusion

UOA testing and urinary metabolomics encompass distinct analytical strategies and clinical applications. Established diagnostic testing for inherited metabolic disorders should be distinguished from commercial preventive panels and targeted or untargeted research workflows. Evidence for analytical performance, clinical validity, and clinical utility is assay- and application-specific; findings from one approach cannot automatically validate another. The aging-related findings support further investigation, but do not establish that UOA-guided assessment or intervention preserves function or improves healthy-aging outcomes.

UOA profiling provides a non-invasive approach to investigating metabolic variation and, when integrated with clinical and complementary omics data, may contribute to understanding metabolic health and aging. Selected metabolite associations and broader urinary metabolomic models support further investigation, although their interpretation remains specific to the assay, population, and outcome studied. Their use to identify subclinical dysfunction, guide individualized prevention, or monitor metabolic resilience remains investigational. Translation into routine care requires independent validation and evidence that test-guided decisions improve clinically meaningful outcomes.

## CRediT authorship contribution statement

**Markus Schauer:** Writing – review & editing, Writing – original draft, Visualization, Validation, Supervision, Resources, Project administration, Methodology, Conceptualization. **Susanne Mair:** Writing – review & editing, Writing – original draft, Supervision. **Mohamad Motevalli:** Writing – review & editing, Writing – original draft, Visualization, Methodology, Conceptualization. **Florian Kainz:** Writing – review & editing, Supervision. **Katharina Wirnitzer:** Writing – review & editing, Writing – original draft, Supervision, Project administration, Methodology, Conceptualization.

## Informed consent statement

Not applicable.

## Institutional review board statement

Not applicable.

## Data availability statement

No new experimental data were generated for this review. The literature search strategies and retrieval counts are provided in Supplementary Table S1. Study-selection procedures and aggregate counts are reported in the Methods, and extracted study-level evidence is presented in Table 3.

## Funding

This research did not receive any funding or grants from agencies in the public, commercial, or non-profit sectors.

## Conflicts of interest

The authors declare no conflicts of interest.
