# Supplementary material for: Urinary organic acid analysis in preventive health assessment and healthy-aging research: A state-of-the-art review of evidence and methodological limitations
Source: Metabol Open. 2026 Oct 15;32:100511. doi: 10.1016/j.metop.2026.100511 (PMC13717086; doi:10.1016/j.metop.2026.100511)
Supplement: Supplementary file 1 [file mmc1.docx]

Supplementary Materials

**Table S1.** Eight complementary PubMed search strategies and retrieval counts.

| **Search and focus** | **Exact query** | **n** |
| --- | --- | --- |
| S1 Organic acid testing and adult preventive applications | (urine[tiab] OR urinary[tiab]) AND ("organic acid"[tiab] OR "organic acids"[tiab]) AND ((aging[tiab] OR ageing[tiab] OR aged[tiab] OR elderly[tiab] OR "older adults"[tiab] OR longevity[tiab] OR centenarian*[tiab] OR frailty[tiab] OR frail[tiab] OR sarcopenia[tiab] OR "biological age"[tiab]) OR (obesity[tiab] OR obese[tiab] OR "metabolic syndrome"[tiab] OR "insulin resistance"[tiab] OR prediabet*[tiab] OR "type 2 diabetes"[tiab] OR "metabolic health"[tiab] OR "cardiometabolic"[tiab]) OR adult*[tiab] OR prevent*[tiab] OR nutrition*[tiab] OR mitochondrial[tiab] OR micronutrient*[tiab] OR vitamin*[tiab]) | 658 |
| S2 Urinary metabolomics and aging | (urine[tiab] OR urinary[tiab]) AND (metabolom*[tiab] OR metabonom*[tiab] OR "metabolic profiling"[tiab] OR "metabolic profiles"[tiab]) AND (aging[tiab] OR ageing[tiab] OR aged[tiab] OR elderly[tiab] OR "older adults"[tiab] OR longevity[tiab] OR centenarian*[tiab] OR frailty[tiab] OR frail[tiab] OR sarcopenia[tiab] OR "biological age"[tiab]) | 389 |
| S3 Urinary metabolomics and cardiometabolic health | (urine[tiab] OR urinary[tiab]) AND (metabolom*[tiab] OR metabonom*[tiab] OR "metabolic profiling"[tiab] OR "metabolic profiles"[tiab]) AND (obesity[tiab] OR obese[tiab] OR "metabolic syndrome"[tiab] OR "insulin resistance"[tiab] OR prediabet*[tiab] OR "type 2 diabetes"[tiab] OR "metabolic health"[tiab] OR "cardiometabolic"[tiab]) | 754 |
| S4 Named organic acids and aging or metabolic health | (urine[tiab] OR urinary[tiab]) AND (hippurat*[tiab] OR citrate[tiab] OR succinate[tiab] OR fumarate[tiab] OR malate[tiab] OR methylmalon*[tiab] OR "alpha-ketoglutarate"[tiab] OR "2-oxoglutarate"[tiab] OR "tricarboxylic acid"[tiab]) AND ((aging[tiab] OR ageing[tiab] OR aged[tiab] OR elderly[tiab] OR "older adults"[tiab] OR longevity[tiab] OR centenarian*[tiab] OR frailty[tiab] OR frail[tiab] OR sarcopenia[tiab] OR "biological age"[tiab]) OR (obesity[tiab] OR obese[tiab] OR "metabolic syndrome"[tiab] OR "insulin resistance"[tiab] OR prediabet*[tiab] OR "type 2 diabetes"[tiab] OR "metabolic health"[tiab] OR "cardiometabolic"[tiab])) | 572 |
| S5 Urinary multi-omics and relevant human phenotypes | (urine[tiab] OR urinary[tiab]) AND ((metabolom*[tiab] OR metabonom*[tiab] OR "metabolic profiling"[tiab] OR "metabolic profiles"[tiab]) OR ("organic acid"[tiab] OR "organic acids"[tiab])) AND (genom*[tiab] OR proteom*[tiab] OR microbiom*[tiab] OR "multi-omic"[tiab] OR "multi-omics"[tiab] OR multiomic*[tiab]) AND (human*[tiab] OR adult*[tiab] OR cohort*[tiab] OR (aging[tiab] OR ageing[tiab] OR aged[tiab] OR elderly[tiab] OR "older adults"[tiab] OR longevity[tiab] OR centenarian*[tiab] OR frailty[tiab] OR frail[tiab] OR sarcopenia[tiab] OR "biological age"[tiab]) OR (obesity[tiab] OR obese[tiab] OR "metabolic syndrome"[tiab] OR "insulin resistance"[tiab] OR prediabet*[tiab] OR "type 2 diabetes"[tiab] OR "metabolic health"[tiab] OR "cardiometabolic"[tiab])) | 571 |
| S6 Urine analytical and preanalytical methods | (urine[tiab] OR urinary[tiab]) AND ((metabolom*[tiab] OR metabonom*[tiab] OR "metabolic profiling"[tiab] OR "metabolic profiles"[tiab]) OR ("organic acid"[tiab] OR "organic acids"[tiab])) AND (normalization[tiab] OR normalisation[tiab] OR preanalytical[tiab] OR "pre-analytical"[tiab] OR "quality control"[tiab] OR "quality assurance"[tiab] OR "sampling protocol"[tiab] OR "sample collection"[tiab] OR "renal handling"[tiab] OR reproducibility[tiab] OR "reference intervals"[tiab]) | 493 |
| S7 Validated supplementary MeSH search | ("Urine"[Mesh] OR "urine"[Subheading]) AND ("Metabolomics"[Mesh] OR "organic acid"[tiab] OR "organic acids"[tiab]) AND ("Aging"[Mesh] OR "Healthy Aging"[Mesh] OR "Frailty"[Mesh] OR "Metabolic Syndrome"[Mesh] OR "Insulin Resistance"[Mesh] OR "Obesity"[Mesh]) | 126 |
| S8 Longitudinal urinary metabolite evidence | (urine[tiab] OR urinary[tiab]) AND (metabolom*[tiab] OR metabonom*[tiab] OR "metabolic profiling"[tiab] OR metabolite*[tiab]) AND ("incident diabetes"[tiab] OR "incident cardiovascular"[tiab] OR "all-cause mortality"[tiab] OR "healthy aging"[tiab] OR "healthy ageing"[tiab] OR "metabolic age"[tiab]) | 78 |
